# Aldo-Keto Reductase 1C15 Characterization and Protection in Ischemic Brain Injury

**DOI:** 10.3390/antiox12040909

**Published:** 2023-04-11

**Authors:** Tuo Yang, Qianqian Li, George Fadoul, Nour Alraqmany, Milos Ikonomovic, Feng Zhang

**Affiliations:** 1 Department of Neurology, University of Pittsburgh, Pittsburgh, PA 15216, USA; 2 Pittsburgh Institute of Brain Disorders and Recovery, University of Pittsburgh, Pittsburgh, PA 15216, USA; 3 Department of Psychiatry, University of Pittsburgh, Pittsburgh, PA 15216, USA; 4 Geriatric Research Education and Clinical Center, VA Pittsburgh Healthcare System, Pittsburgh, PA 15240, USA

**Keywords:** stroke, ischemic preconditioning, blood–brain barrier, Nrf2, 4-hydroxynonenal, inflammation

## Abstract

Aldo-keto reductase (AKR) 1C15, a member of the AKR superfamily, was recently identified and cloned, and reported to alleviate oxidative stress in endothelial cells in rodent lungs. However, its expression and role in the brain and ischemic brain diseases have not been investigated. AKR1C15 expression was detected with real-time PCR. Mouse ischemic stroke and ischemic preconditioning (IPC) were established with middle cerebral artery occlusion (MCAO) for 1 h or 12 min, respectively. Recombinant AKR1C15 was administered intraperitoneally, and stroke outcome was evaluated with neurobehavioral tests and infarct volumes. Rat primary brain cell cultures were subjected to oxygen–glucose deprivation (OGD) to mimic ischemic injury. Cell survival or in vitro blood–brain barrier (BBB) permeability was measured, and nitric oxide (NO) release was detected. Immunostaining and Western blotting were used to evaluate oxidative-stress-related protein expression. AKR1C15 administration decreased the infarct volume and neurological deficits 2d post-stroke, and its early (1-h) administration after IPC abolished the protection of IPC against stroke. In rat primary brain cell cultures, AKR1C15 was most abundantly expressed in brain microvascular endothelial cells (BMVECs) and microglia. Its expression decreased upon OGD in most cell types except for BMVECs and microglia. In primary neuronal cultures, AKR1C15 treatment prevented OGD-induced cell death accompanied by decreased levels of 4-hydroxynonenal, 8-hydroxy-2′-deoxyguanosine, and heme oxygenase-1. In BMVEC cultures, AKR1C15 treatment protected against OGD-induced cell death and in vitro BBB leakage. In primary microglial cultures, AKR1C15 reduced the release of NO upon proinflammatory stimulation. Our results provide a characterization of the novel antioxidant AKR1C15 and demonstrate its protective role against ischemic injury, both in vivo and in vitro. AKR1C15 may be a promising agent for ischemic stroke treatment.

## 1. Introduction

Ischemic stroke represents a leading cause of death and long-term disability nationwide [1]. Since the brain is rich in lipid contents, lipid electrophiles, such as 4-hydroxynonenal (4-HNE)—the product of omega-6 polyunsaturated fatty acid peroxidation—are constitutively generated, which are an important form of cell signaling under physiological conditions [2,3]. In particular, subthreshold levels of 4-HNE could be beneficial and protective against subsequent lethal damage, serving as an important mechanism underlying ischemic preconditioning (IPC)-mediated neuroprotection [4,5]. However, after ischemic stroke, excessive oxidative stress results in overwhelming lipid peroxidation and a pathological accumulation of lipid electrophiles [6,7], which are detrimental to cells because they cause DNA damage and acute inflammation before ultimately causing cell death through necrosis or apoptosis [8,9]. Thus, reducing 4-HNE and other lipid electrophiles may be a promising strategy for neuroprotection in the acute phase of ischemic stroke.

Aldo-keto reductase (AKR) is a large superfamily consisting of more than 190 members across multiple species [10]. They are powerful reducers and, therefore, may be useful in defense against oxidative injuries. AKR1C subfamily has recently emerged as a hot topic given its capability to modify lipids, such as steroids and prostaglandins [11,12,13]. AKR1C15, the AKR1C subfamily member found in *Rattus*, was identified and cloned [14]. Excitingly, in endothelial cultures, AKR1C15 has been reported to alleviate cell injury induced by 4-HNE and oxidized low-density lipoprotein [15]. AKR1C15 is also present in the brain, albeit at a low level compared to other organs such as the lung, the stomach, and the colon [16]. However, its expression in different types of brain cells and whether it might protect against ischemic stroke have yet to be investigated.

In the present article, we report for the first time that the exogenous administration of recombinant AKR1C15 is protective in a mouse ischemic stroke model, while abolishing IPC-afforded protection. We characterize the expression of AKR1C15 in primary cultures of central nervous system (CNS) cells and demonstrate that AKR1C15 protects neurons and brain microvascular endothelial cells (BMVECs) and attenuates microglial inflammatory responses. These observations shed additional light on the mechanisms underlying AKR1C15-mediated neuroprotection.

## 2. Materials and Methods

### 2.1. Generation and Quality Control of TAT-AKR1C15 Fusion Protein

**Figure 1:**
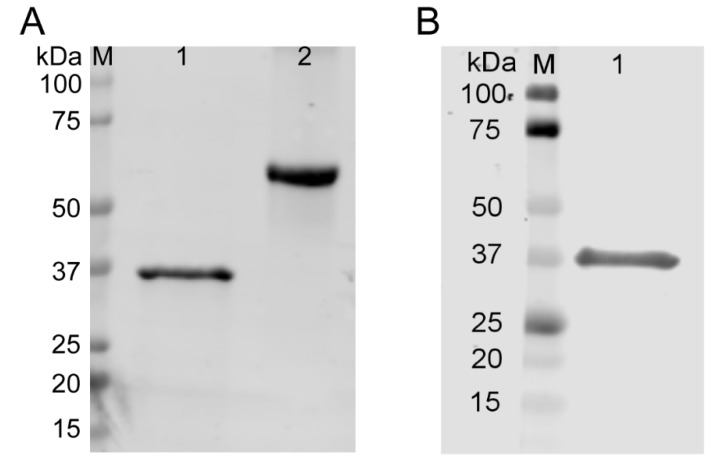
Validation of TAT-AKR1C15 fusion protein. TAT-AKR1C15 protein with His tag synthesized by Biomatik was subjected to electrophoresis and transferred onto the polyvinylidene difluoride (PVDF) membrane. (**A**) Total protein staining. M: marker; Lane 1: TAT-AKR1C15; Lane 2: bovine serum albumin (BSA). (**B**) Western blotting with anti-His antibody. M: marker; Lane 1: TAT-AKR1C15.

The transactivator of transcription (TAT) peptide (GRKKRRQRRRPQ) is derived from the human immunodeficiency virus exerting a cell-penetrating property [17,18]. TAT-AKR1C15 fusion protein harboring a His tag was biosynthesized by Biomatik (Wilmington, DE, USA) and validated (Figure 1).

### 2.2. Animals and Drug Administration

All animal experiments were approved by the University of Pittsburgh Institutional Animal Care and Use Committee (approval numbers of protocols: 18042610 and 21069543) and performed following the National Institutes of Health Guide for the Care and Use of Laboratory Animals following the Stroke Therapy Academic Industry Roundtable (STAIR) recommendations [19]. Adult male C57BL/6J mice (age: 10–12 weeks old) were obtained from Jackson Laboratory (Bar Harbor, ME, USA). The mice were housed in a temperature- and humidity-controlled animal facility with a 12:12 h light–dark cycle. Food and water were available *ad libitum*. Every attempt was made to limit animal suffering and the quantity of animals killed.

For AKR1C15 administration, TAT-AKR1C15 fusion protein or vehicle (Veh) was injected intraperitoneally (i.p.) at indicated doses after stroke or IPC. All the outcome assessments were performed by investigators blinded to the group assignments.

### 2.3. Middle Cerebral Artery Occlusion (MCAO)

Mice underwent MCAO on the left side for 12 min to induce IPC and 60 min to induce ischemic stroke, as previously reported [4,5,20]. These procedures were followed by reperfusion for the indicated durations. The interval between IPC and stroke was 3 days.

MCAO was performed with standard, previously published procedures [4,5,6,18]. In brief, mice were anesthetized with 1.5% isoflurane in a 30% O_2_/70% N_2_O mixture under spontaneous breathing, and rectal temperature was maintained at 37.0 ± 0.5 °C with a temperature-regulated heating pad. Mean arterial blood pressure was monitored with a tail-cuff and maintained at 90 ± 5 mmHg across all groups. Under a surgical microscope, the left external, internal, and common carotid arteries were exposed through a midline neck incision. After coagulating and cutting the branches of the external carotid arteries, a 7–0 monofilament nylon suture with a silicone coat was inserted in the lumen of the external carotid artery and advanced to the origin of the middle cerebral artery via the internal carotid artery. Regional cerebral blood flow (CBF) was measured using laser Doppler flowmetry, and the success of ischemia was confirmed by the measurement of CBF and the examination of neurological deficits [5]. Mice were excluded from study if the CBF failed to decrease to 25% of baseline during ischemia or failed to recover to 80% during reperfusion. CBF was monitored for 5 min during the reperfusion phase.

### 2.4. Neurological Scoring

Neurologic dysfunction was scored at indicated time points after MCAO using the 5-point method as previously described [6,21], with 0 being the least dysfunction and 4 the worst dysfunction (Table 1).

### 2.5. Measurement of Infarct Volume

Infarct volumes were measured as described previously using 2,3,5-triphenyl tetrazolium chloride (TTC) staining [5]. The brains were removed 2 days after stroke and sliced into seven 1 mm thick coronal sections. Sections were immersed in prewarmed 2% TTC (Sigma-Aldrich, St. Louis, MO, USA) in saline for 10 min and then fixed in 4% paraformaldehyde (PFA; Sigma-Aldrich, St. Louis, MO, USA). Infarct volume was measured by a blinded observer using National Institutes of Health ImageJ software (Version: 1.53k) and was corrected for brain edema by reporting the volume of the contralateral hemisphere minus the noninfarcted volume of the ipsilateral hemisphere.

### 2.6. Primary Cell Cultures

Sprague Dawley rat primary cortical neuronal cultures were performed as previously described [4,22]. In brief, cortices from E16–18 fetuses were dissected, trypsinized, and filtered through a 40 µm strainer to prepare a single cortical cell suspension. Cortical cells were seeded onto tissue culture plates precoated with poly-D-lysine (PDL, Sigma Aldrich, St. Louis, MO, USA) and maintained in neurobasal media supplemented with B27 (both from Gibco, ThermoFisher, Pittsburgh, PA, USA) for 10–14 days.

Microglia, oligodendrocyte precursor cell (OPC), and astrocyte cultures were derived from mixed glial cell cultures prepared using the brains of 1-day-old Sprague Dawley rat pups [23]. Brain cortical tissues were prepared into single cells and cultured in poly-D-lysine (PDL)-coated T175 flasks filled with culture media (Dulbecco’s Modified Eagle Medium (DMEM)/F12 containing 10% fetal bovine serum (FBS), 2 mM _L_-glutamine, 1 mM sodium pyruvate, 100 mM nonessential amino acids, 50 U/mL penicillin, and 50 mg/mL streptomycin). The medium was changed twice a week. After 12–14 days, microglia were detached by shaking the flasks for 1 h at 180 r/min. Following this, microglia were harvested and seeded in PDL-coated plates and incubated for 1 day before use. In the meantime, the flasks were refilled with fresh media and were subjected to shaking at 200 r/min overnight to separate OPCs from the firmly attached astrocyte layer. OPCs were maintained for 3 days in a serum-free basal defined medium (DMEM, 0.1% bovine serum albumin, 50 μg/mL human apo-transferrin, 50 μg/mL insulin, 30 nM sodium selenite, 10 nM D-biotin, and 10 nM hydrocortisone) containing 10 ng/mL platelet-derived growth factor and 10 ng/mL basic fibroblast growth factor. After shaking of the OPCs, the bottom layer was astrocytes, which was maintained in DMEM containing 10% FBS.

Primary rat brain microvascular endothelial cells (BMVECs) were purchased from Cell Applications (San Diego, CA, USA) and were maintained in rat brain endothelial cell growth medium consisting of basal medium and appropriate growth factors (Cell Applications, San Diego, CA, USA).

### 2.7. Oxygen–Glucose Deprivation (OGD)

To mimic ischemia, primary cell cultures were exposed to standard oxygen–glucose deprivation (OGD), followed by reoxygenation for 2, 8, or 24 h, as previously described [4,22]. The durations of OGD for each cell type (1 h in neurons, 1.5 h in OPCs, 4 h in astrocytes, 12 h in BMVECs, and 1.5 h in microglia) were determined based on our previous experience that could induce significant cell injury with reduced viability.

To evaluate the role of AKR1C15 after OGD in neurons and BMVECs, vehicle or TAT-AKR1C15 were added during the reperfusion phase at a dose of 10 µg/mL.

### 2.8. Cell Viability and Death Assays

Cell viability was determined via the incubation of cells with 3-(4,5-dimethylthiazol-2-yl)-2,5-diphenyltetrazolium bromide (MTT, Sigma Aldrich) at 5 mg/mL for 30 min while purple crystals were visible under a microscope. After a brief wash, dimethyl sulfoxide was added and the absorbance at 595 nm was read by a spectrophotometer. Data were expressed as a percentage of the control group. Extracellular lactate dehydrogenase (LDH) released from damaged cells was measured with an LDH detection kit (Pointe Scientific Inc, Canton, MI, USA) according to the manufacturer’s instructions. The findings were expressed as a relative percentage in relation to the control group’s LDH release from a whole cell lysate.

### 2.9. In Vitro Blood–Brain Barrier Model and Permeability Assay

In vitro BBB models were established to measure permeability, as previously described [5,24]. Transwell inserts (pore size 0.4 µm, Falcon, Corning, NY, USA) were placed into 24-well plates to divide each well into top (luminal) and bottom (abluminal) compartments. This apparatus allows for the free passage of nutrients while cellular trafficking across the compartments is blocked. After OGD treatment, fluorescein 5-isothiocyanate-dextran (4 kDa, Sigma-Aldrich, St. Louis, MO, USA) was added into the top compartment at a final concentration of 0.05 mg/mL for the 6 h, and fluorescence intensity was measured in an aliquot of 50 µL of media from the bottom compartment.

### 2.10. Nitric Oxide (NO) Release Assay

Microglial cultures were exposed to 100 ng/mL lipopolysaccharides (LPS, from *E. coli*, Sigma Aldrich, St. Louis, MO, USA) for 24 h in the presence or absence of TAT-AKR1C15 at indicated doses, and supernatants from microglial cultures were collected. NO in the supernatants was measured with Griess Reagent (Cell Signaling Technology, Boston, MA, USA) following the manufacturer’s instructions.

### 2.11. Real-Time PCR (RT-PCR)

Total RNA was extracted from rat cell cultures using the RNeasy kit (QIAGEN, Germantown, MD, USA). First-strand cDNA was synthesized with the iScript cDNA synthesis kit (Bio-Rad, Hercules, CA, USA) according to the manufacturer’s protocol. The program for reverse transcription was 5 min at 25 °C, 20 min at 46 °C, 1 min at 95 °C, and maintained at 4 °C. PCR was performed on the Opticon 2 Real-Time PCR Detection System (Bio-Rad) using primers (Table 2) and iTaq University SYBR Green Supermix (Bio-Rad). The program for real-time PCR was 30 s at 95 °C, (5 s at 95 °C, 30 s at 60 °C) × 40 cycles. The cycle time values were normalized to Gapdh in the same sample as an internal control. In one batch of the experiments, the TAT-AKR1C15 plasmid (Biomatik) was used as a reference to compare Akr1c15 expression among different cell types.

### 2.12. Immunocytochemistry

Neuronal cultures were washed with phosphate buffered saline (PBS) and fixed with 4% PFA in PBS. After blocking with 5% donkey serum (Jackson ImmunoResearch Laboratories, West Grove, PA, USA), cells were incubated with primary antibodies including anti-4-hydroxynonenal (4-HNE, 1:500; R&D Systems, Cat# MAB3249, Minneapolis, MN, USA) and anti-8-hydroxydeoxyguanosine (8-OH-dG, 1:500, Millipore, Cat# AB5830, Burlington, MA, USA) at 4 °C overnight. After washing, cells were incubated for 1 h at room temperature with donkey secondary antibodies conjugated with Alexa Fluor 488 or Cy3 (1:1000; Jackson ImmunoResearch Laboratories, West Grove, PA, USA). The cells were then stained with Hoechst (ThermoFisher, Pittsburgh, PA, USA) and images were taken with an EVOS microscope. The analysis of the images was performed using the ImageJ software (Version: 1.53k) by an investigator blinded to the experimental group assignments. Data are expressed as the numbers of 4-HNE^+^ or 8-OH-dG^+^ cells over total Hoechst^+^ cells.

### 2.13. Electrophoresis and Western Blot

Whole-cell lysates were prepared with radio immunoprecipitation assay buffer (Cell Signaling Technology, Boston, MA, USA). Protein concentrations were determined using the Bradford protein assay (Bio-Rad, Hercules, CA, USA). Equal amounts of protein samples were loaded onto acrylamide gels and subjected to electrophoresis, followed by transfer to polyvinylidene difluoride (PVDF) membranes (Millipore, Burlington, MA, USA). Total protein staining was performed with a Revert Total Protein Staining Kit (LI-COR, Lincoln, NE, USA). PVDF membranes were then blocked with 5% nonfat milk and probed with antibodies recognizing His tag (1:2000, Novus Biologicals, Cat# NBP125939, Centennial, CO, USA), heme oxygenase-1 (HO-1, 1:1000, Enzo Life Sciences, Cat# ADI-SPA-896, Farmingdale, NY, USA), and β-Actin (1:3000, Sigma-Aldrich, Cat# A2228, St. Louis, MO, USA). After incubation in secondary antibodies (Cell Signaling) for 1 h, the membranes were incubated with ECL substrates (Pierce, ThermoFisher, Pittsburgh, PA, USA) and developed with X-ray film. ImageJ software (Version: 1.53k) was used for gel analyses.

### 2.14. Statistical Analyses

All data are presented as mean ± standard deviation (SD) unless indicated otherwise. The statistical differences among the means of multiple groups were assessed with a one-way or two-way ANOVA followed by a post hoc Tukey’s multiple comparison test. A *p*-value of less than 0.05 was deemed statistically significant.

## 3. Results

### 3.1. AKR1C15 Reduced Infarct Volumes and Neurological Deficits after Ischemic Stroke

**Figure 2:**
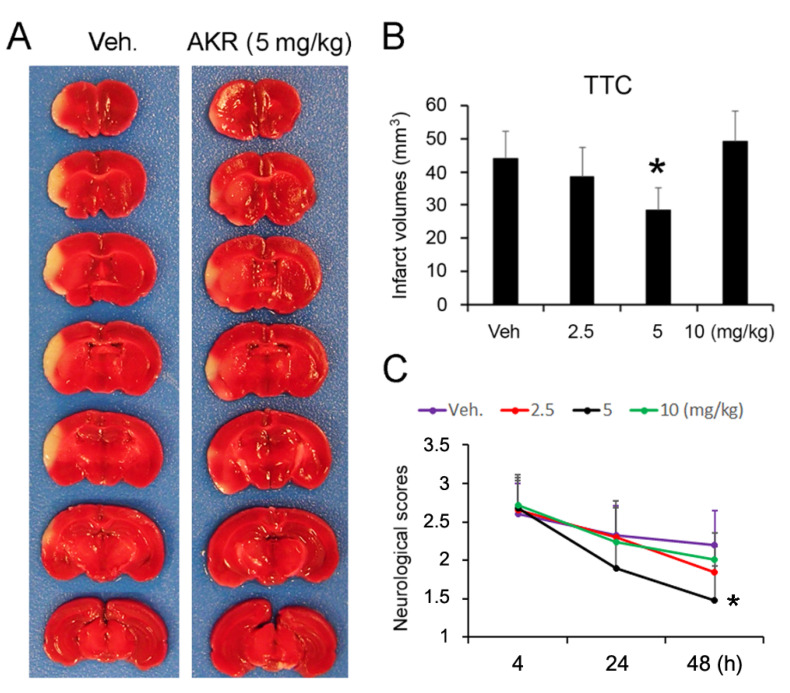
AKR1C15 reduced infarct volumes and neurological deficits after MCAO. TAT-AKR1C15 or vehicle (Veh.) was i.p. injected at indicated doses right after MCAO. (**A**) Representative photographs of TTC-stained brain sections and (**B**) calculated infarct volumes at 48 h after MCAO. Data are mean ± SD; *n* = 7–9 animals per group. One-way ANOVA followed by post hoc Tukey’s multiple comparison. (**C**) Neurological scores at 48 h after MCAO. Two-way ANOVA followed by post hoc Tukey’s multiple comparison. Data are mean ± SD; *n* = 7–9 animals per group. * *p* < 0.05 vs. Veh.

Given the robust potency of AKR1C15 as an electrophile scavenger and its powerful reducing capacity, we hypothesized that it would exert protection against ischemic stroke. We, therefore, evaluated its effect on infarct volume and neurological scores up to 48 h after ischemic stroke. We observed that 48 h after i.p. administration of 5 mg/kg TAT-AKR1C15, the infarct volumes were significantly reduced (Figure 2A,B), and the neurological deficits were alleviated (Figure 2C). These results imply that AKR1C15 may be a novel compound that can protect against acute ischemia brain injury.

### 3.2. Early Injection of AKR1C15 Abolished the Protective Role of IPC

**Figure 3:**
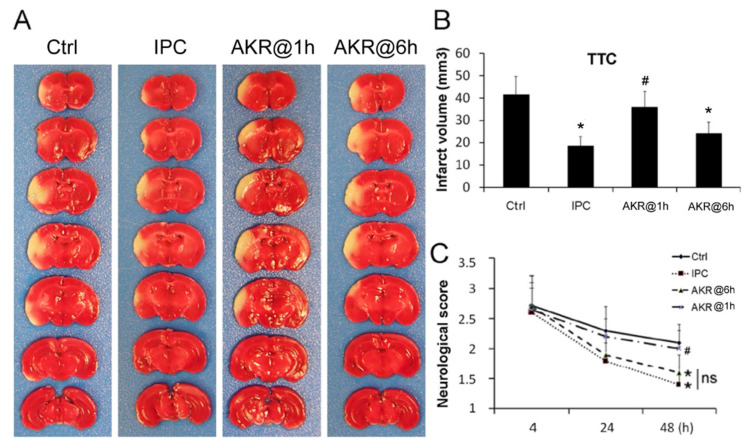
Early injection of AKR1C15 abolished the protective role of IPC. At 1 h or 6 h after IPC, mice were i.p. injected with TAT-AKR1C15 at 5 mg/kg, and MCAO was performed 3 days after IPC. (**A**) Representative photographs of TTC-stained brain sections and (**B**) calculated infarct volumes at 48 h post-MCAO. Data are mean ± SD; *n* = 7–9 animals per group. One-way ANOVA followed by post hoc Tukey’s multiple comparison. (**C**) Neurological scores at 48 h after MCAO. Two-way ANOVA followed by post hoc Tukey’s multiple comparison. Data are mean ± SD; *n* = 7–9 animals per group. * *p* < 0.05 vs. Ctrl. # *p* < 0.05 vs. IPC. ns: not significant.

Previous work from our group on the mechanisms underlying IPC-afforded protection against ischemic stroke has revealed that lipid electrophiles such as 4-HNE are generated after IPC [4], which is essential as the neutralization of electrophiles by thiol antioxidant *N*-acetyl cysteine completely abolished IPC-mediated neuroprotection [5]. Because AKR1C15 has been described as a powerful electrophile scavenger, we sought to see if it could abolish IPC-provided protection. To this end, TAT-AKR1C15 was i.p. administered at 1 h (early) and 6 h (late) after IPC. As shown in Figure 3, early injection abolished the IPC protection against both infarct formation and neurological damage, while late injection when electrophiles already exerted their function failed to do so. These findings validate that AKR1C15 is a powerful electrophile scavenger and provide another line of evidence on the critical role of electrophiles for IPC-afforded protection against ischemic stroke.

### 3.3. AKR1C15 Expression in Primary CNS Cell Cultures before and after OGD

**Figure 4:**
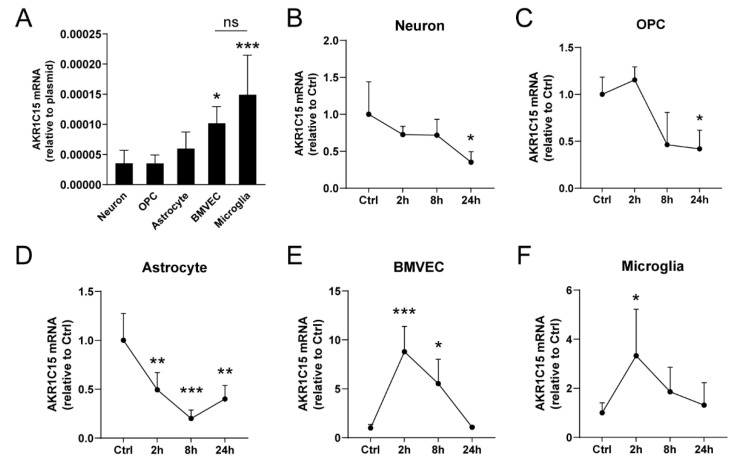
AKR1C15 expression in rat primary CNS cell cultures before and after OGD. AKR1C15 mRNA in primary rat CNS cell cultures was determined by real-time PCR. (**A**) Baseline AKR1C15 expression levels compared to TAT-AKR1C15 plasmid (Biomatik). Data are mean ± SD. *n* = 6–9 independent experiments. One-way ANOVA followed by Tukey’s multiple comparison. *, *** *p* < 0.05, 0.001 vs. Neuron. ns: not significant. Cell cultures were then subjected to OGD/reoxygenation, and AKR1C15 mRNA expressions were detected at 2, 8, and 24 h after reoxygenation in cultured (**B**) neurons (1 h OGD), (**C**) OPCs (90 min OGD), (**D**) astrocytes (4 h OGD), (**E**) BMVECs (12 h OGD), and (**F**) microglia (90 min OGD). Data are mean ± SD. *n* = 4–9 independent experiments. One-way ANOVA followed by Tukey’s multiple comparison. *, **, *** *p* < 0.05, 0.01, 0.001 vs. Ctrl. OGD: oxygen–glucose deprivation. BMVEC: brain microvascular endothelial cell. OPC: oligodendrocyte precursor cell.

We then sought to determine the underlying mechanisms for AKR1C15 to protect against ischemic stroke, starting from the detection of its expression in different CNS cell types. Previous study reported a low level of AKR1C15 mRNA expression in the brain tissue compared to the lung, colon, and stomach in rats [16]. We then tested the AKR1C15 mRNA levels in primary rat cell cultures under naïve conditions and found that it was most abundantly expressed in BMVECs and microglia (Figure 4A).

We then tested the expression profile of AKR1C15 mRNA after OGD in each cell type. The duration of OGD was determined based on our experience with each cell type and our OGD model, where this duration typically leads to significant cell death, to a degree that is partially rescuable. We noticed significantly decreased expression levels of AKR1C15 with time in primary neurons, OPCs, and astrocytes. On the other hand, BMVECs and microglia showed acutely elevated AKR1C15 expression that went back to normal with time.

### 3.4. AKR1C15 Protected Neurons against OGD Associated with Reduced Lipid Electrophile and Oxidative Stress

**Figure 5:**
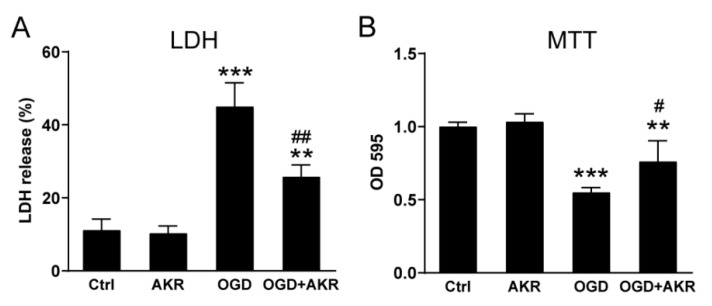
AKR1C15 protected against OGD-induced neuronal death. Primary rat neurons were subjected to 1 h OGD followed by 24 h reoxygenation in the presence or absence of 10 µg/mL TAT-AKR1C15. (**A**) LDH and (**B**) MTT assays were conducted 24 h after reoxygenation. Data are mean ± SD. *n* = 3–6 independent experiments. One-way ANOVA followed by Tukey’s multiple comparison. **, *** *p* < 0.01, 0001 vs. Ctrl. #, ## *p* < 0.05, 0.01 vs. OGD. LDH: lactate dehydrogenase. MTT: 3-(4,5-dimethylthiazol-2-yl)-2,5-diphenyltetrazolium bromide. OGD: oxygen–glucose deprivation.

Neurons are the most important functional cells in the brain. We then explored if AKR1C15 was able to directly rescue OGD-induced neuronal death. Primary rat neuronal cultures were subjected to 1 h of OGD followed by 24 h of reoxygenation in the presence of 10 μg/mL TAT-AKR1C15. We found AKR1C15 successfully rescued OGD-induced neuronal death, as evidenced by decreased LDH in the culture medium (Figure 5A) and increased MTT uptake (Figure 5B), respectively.

**Figure 6:**
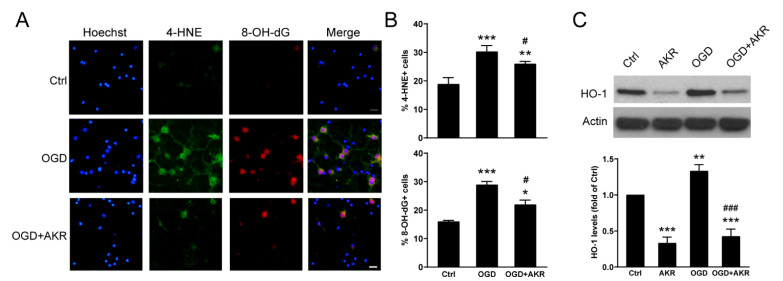
AKR1C15 protected against OGD-induced oxidative stress in neuronal cultures. Primary rat neurons were subjected to 1 h OGD followed by 24 h reoxygenation in the presence or absence of 10 µg/mL TAT-AKR1C15. (**A**) Representative 4-HNE and 8-OH-dG staining and (**B**) cell counting. Scale bar: 20 µm. (**C**) Representative Western blotting and semiquantitation for HO-1 (original Western blotting shown in Figure S1). Data are mean ± SD. *n* = 3–4 independent experiments. One-way ANOVA followed by Tukey’s multiple comparison. *, **, *** *p* < 0.05, 0.01, 0001 vs. Ctrl. #, ### *p* < 0.05, 0.001 vs. OGD. OGD: oxygen–glucose deprivation. 4-HNE: 4-hydroxynonenal. 8-OH-dG: 8-hydroxydeoxyguanosine. HO-1: heme oxygenase-1.

Moving forward, we assessed the amounts of 4-HNE, which represents lipid electrophile production, following OGD. We noticed that AKR1C15 significantly reduced 4-HNE levels induced by OGD (Figure 6A,B), confirming the role of AKR1C15 in eliminating lipid electrophiles. We also examined the level of 8-OH-dG, an indicator of oxidative-stress-related DNA damage [25], and revealed that AKR1C15 also alleviated 8-OH-dG levels induced by OGD, suggesting that AKR1C15 could also protect against oxidative-stress-related DNA damage, which may serve as a mechanism for neuronal protection. Since lipid peroxidation and oxidative stress are associated with compensatory nuclear-factor-erythroid-2-related factor 2 (Nrf2) activation and downstream phase 2 enzyme upregulation [26], we detected the levels of HO-1, a phase 2 enzyme and a reliable marker for Nrf2 activation, in neurons after OGD. Indeed, the upregulation of HO-1 was brought down by AKR1C15 (Figure 6C and Figure S1), suggesting reduced oxidative stress by AKR1C15 treatment, which diminished the necessity of subsequent compensatory Nrf2 activation. Another possible explanation is that AKR1C15 may directly interact with the Nrf2 pathway and regulate its activity.

### 3.5. AKR1C15 Protected BMVECs against OGD-Induced Cell Death and In Vitro BBB Leakage

**Figure 7:**
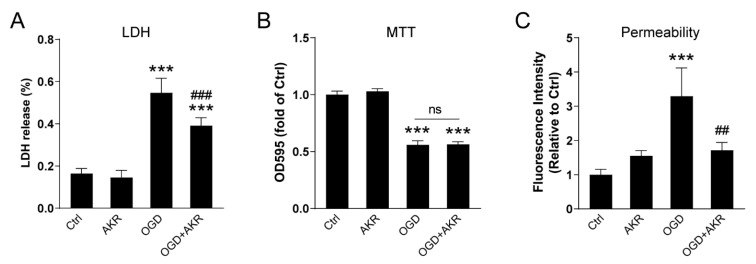
AKR1C15 protected BMVECs against OGD-induced cell death and BBB leakage. Primary rat BMVECs were subjected to 12 h OGD followed by reoxygenation in the presence or absence of 10 µg/mL TAT-AKR1C15. Cell viability and death were evaluated by (**A**) LDH assay and (**B**) MTT assay at 8 h after reoxygenation. In vitro BBB model was applied, and (**C**) permeability was evaluated at 6 h after reoxygenation. Data are mean ± SD. *n* = 4 independent experiments. One-way ANOVA followed by Tukey’s multiple comparison. *** *p* < 0001 vs. Ctrl. ##, ### *p* < 0.01, 0.001 vs. OGD. ns: not significant. BMVEC: brain microvascular endothelial cells. OGD: oxygen–glucose deprivation. LDH: lactate dehydrogenase. MTT: 3-(4,5-dimethylthiazol-2-yl)-2,5-diphenyltetrazolium bromide. BBB: blood–brain barrier.

Since BMVECs had abundant AKR1C15 expression before and after OGD, we were curious if this could be an internal protective mechanism for BMVEC. We, therefore, tested if the addition of AKR1C15 could rescue BMVEC death and preserve BMVEC functions. BMVECs were subjected to 12 h of OGD followed by reoxygenation in the presence of 10 μg/mL TAT-AKR1C15. As expected, AKR1C15 significantly reduced OGD-induced LDH leakage (Figure 7A). However, it failed to rescue cell viability, as evidenced by the MTT assay (Figure 7B), upon OGD. This result indicated that, although AKR1C15 could prevent OGD-induced BMVEC death, it may not be sufficient to completely restore the metabolic activity in BMVECs.

To further evaluate BMVEC function, an in vitro BBB model was established as previously reported [5,24]. After 12 h of OGD, the in vitro BBB was reoxygenated in the presence of 10 μg/mL TAT-AKR1C15, and fluorescence intensities in the abluminal compartments were measured. As expected, AKR1C15 significantly reduced the leakage of the fluorescent dye, suggesting its preservation of in vitro BBB functioning.

### 3.6. AKR1C15 Reduced Microglial NO Release upon Proinflammatory Stimulation

**Figure 8:**
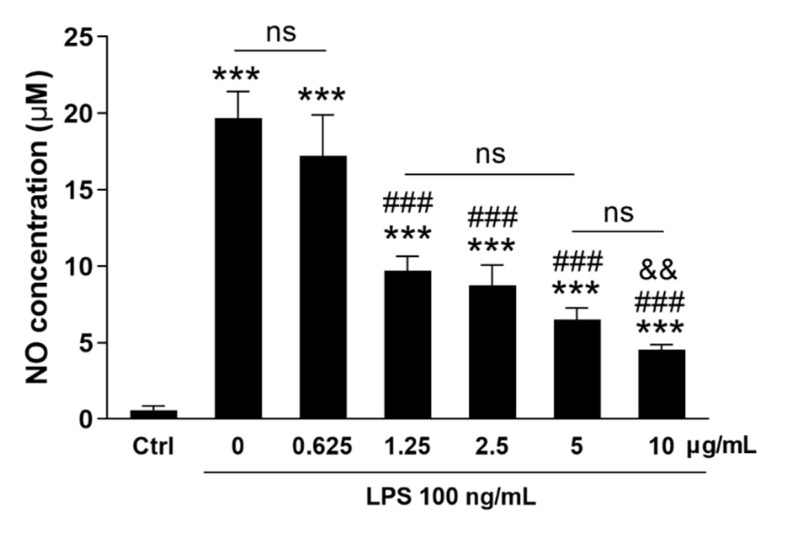
AKR1C15 reduced LPS-induced NO release in microglial cultures. Primary rat microglial cultures were treated with 100 ng/mL LPS in the presence of TAT-AKR1C15 at indicated doses for 24 h, and NO concentration in the culture media was measured. Data are mean ± SD. *n* = 4–6 independent experiments. One-way ANOVA followed by Tukey’s multiple comparison. *** *p* < 0001 vs. Ctrl. ### *p* < 0.001 vs. 0 and 0.625 µg/mL groups. && *p* < 0.01 vs. 1.25 and 2.5 µg/mL groups. ns: not significant. LPS: lipopolysaccharides. NO: nitric oxide.

Microglial proinflammatory phenotypes are associated with worsened stroke outcomes [27,28]. To this end, we explored whether AKR1C15 could alter or mitigate pro-inflammatory responses upon LPS, a traditional inducer of the proinflammatory phenotype in myeloid cells [29,30]. Interestingly, LPS-induced NO release could be significantly reduced by TAT-AKR1C15 in a dose-dependent manner (Figure 8), confirming the anti-inflammatory effect of AKR1C15, which may serve as another layer of the mechanism underlying its protection against ischemic stroke.

## 4. Discussion

In the present study, we examined the effect of AKR1C15 in stroke and IPC and reported the expression profile of AKR1C15 in primary CNS cell cultures before and after OGD. We also confirmed that AKR1C15 is protective in cultured neurons and BMVECs, and it exerts anti-inflammatory properties in cultured microglia. The study is novel in several ways: (1) it is the first to study AKR1C15 in the context of stroke, (2) it describes the respective AKR1C15 expression profile in different CNS cells for the first time, and (3) it is the first to explore the effect of AKR1C15 in primary CNS cell cultures.

AKR is a large superfamily with a molecular weight of approximately ~37 kDa (Figure 1A,B), which catalyzes the NAD(P)H-dependent reduction of carbonyl groups and has a wide variety of substrates [31,32]. The AKR1C subfamily is well known for its lipid modification activity, and AKR1C15 piqued our interest due to its strong ability to remove lipid electrophiles such as 4-HNE [15]. It is worth noting that AKR1C isoforms vary from species to species. For example, AKR1C1-4 are found in *Homo*, AKR1C15-17 are found in *Rattus*, while AKR1C12-14 and 19–21 are found in *Mus* [10]. In the current study, we injected the AKR1C15, a rat isoform, into mice as an exogenous compound. Because AKR isoforms function differently, and there may not be homology among different species [10,33], one needs to be cautious when applying findings from one species to another.

The present study is the first in the field to apply AKR1C15 in the context of ischemic stroke. The rationale for this approach stems from reports that 4-HNE plays a role in ischemic brain injury and that AKR1C15 rescued 4-HNE-induced endothelial damage [15]. The effect of 4-HNE in stroke is complicated as a double-edged sword. On one hand, excessive 4-HNE is detrimental and leads to cell death [34]. On the other hand, sublethal 4-HNE is critical for IPC and neuroprotection through triggering internal antioxidant defense mechanisms [4,5]. As a result, delicate dose adjustment for 4-HNE may be warranted to protect the brain without triggering significant injury [35]. In concert with this, AKR1C15, the robust 4-HNE scavenger, protected against ischemic injury while simultaneously reducing IPC-afforded neuroprotection. Consistently, in neuronal cultures, AKR1C15 reduced OGD-mediated 4-HNE production and subsequent oxidative DNA injury, and simultaneously reduced antioxidant defense mechanisms as evidenced by decreased HO-1 production. This may partially explain our finding that only a medium dose (5 mg/kg) of AKR1C15 could reduce infarct volume and neurological deficits (Figure 2), whereas both low dose (2.5 mg/kg) and high dose (10 mg/kg) failed to do so—low dose may not be sufficient to scavenge lipid electrophiles, while high dose abolished the internal antioxidant defense. We did not include female mice in the present study, mainly because some AKRs, for example AKR1C3, can increase estrogen levels [36], which could potentially confound the protective effects afforded by AKRs.

We also examined the AKR expression profile in a panel of CNS cells at baseline or after OGD. A previous study reported that 4-HNE could trigger AKR1C15 expression in rat prostate endothelial cell cultures [15]. Consistently, we observed elevated AKR1C15 expression in BMVECs, as well as microglia, indicating 4-HNE production caused by oxidative stress may be promoting AKR1C15 expression. Interestingly, among a panel of CNS cells, only BMVECs and microglia displayed increased AKR1C15 expression upon OGD. A possible explanation is that BMVECs are the most proliferative cell type in the brain, with high transcriptional activity which corresponds well to their proliferative and regenerative property, and microglia are highly dynamic myeloid cells which actively undergo phenotype shifting through changing their transcriptomics upon ischemic insult. We also confirmed that OGD-induced cell injury could be alleviated by AKR1C15 in both neurons and BMVECs, and that microglial proinflammatory responses could be suppressed by AKR1C15. It is worth noting that these mechanisms were tested in in vitro cell cultures, which may not completely represent the in vivo situation. Future studies should apply in vivo approaches to validate the findings.

Our study has some limitations. For instance, AKR1C15 detection was at the RNA level since, currently, there are no commercially available AKR1C15 antibodies. Secondly, we did not examine long-term outcomes in our stroke model. Lipid electrophiles are labile, nonstable molecules that affect the acute phase more than the chronic phase. Nevertheless, it will be important to explore how acute-phase events and interventions can affect long-term outcomes after ischemic stroke.

## 5. Conclusions

In summary, we did AKR1C15 characterization after ischemic stroke and demonstrated its protective role against ischemic injury both in vivo and in vitro. AKR1C15 may be a promising agent for ischemic stroke treatment.

## Figures and Tables

**Table 1:** Neurological scoring system.

Score	Criteria
0	No neurologic deficit.
1	Mild focal neurologic deficit. Failure to fully extend left forepaw.
2	Moderate focal neurologic deficit. Circling to the left.
3	Severe focal neurologic deficit. Falling to the left.
4	Could not walk spontaneously and with decreased level of consciousness.

**Table 2:** Primers used in the study.

mRNA	Forward	Reverse	Accession Number
Akr1c15	GATACTTGGGAGGCACTGGA	TGGTTGAGATACGGGTGACA	NM_001109900.1
Gapdh	TGTTGCCATCAACGACCCCTT	CTCCACGACATACTCAGCA	NM_017008.4

## Data Availability

The data presented in this study are available in the article and Supplementary Material.

## Supplementary Materials

The following supporting information can be downloaded at: https://www.mdpi.com/article/10.3390/antiox12040909/s1, Figure S1: Whole blot for Western blot images in Figure 6C. Rectangles present areas cropped in Figure 6C.
