# Supplementary material for: Aldo-Keto Reductase 1C15 Characterization and Protection in Ischemic Brain Injury
Source: Antioxidants (Basel). 2023 Apr 11;12(4):909. doi: 10.3390/antiox12040909 (PMC10135333; doi:10.3390/antiox12040909)
Supplement: Supplementary file 1 [file antioxidants-12-00909-s001.zip › antioxidants-2262732-supplementary.pdf]

Supplementary Material

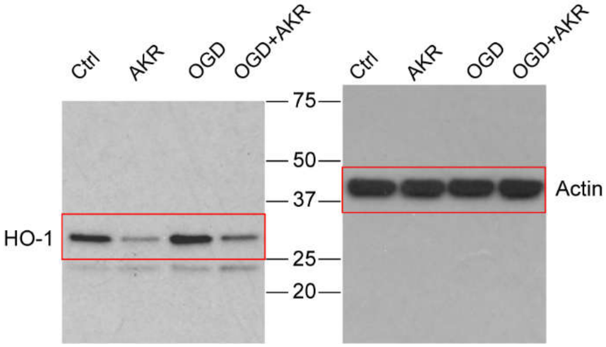

Figure S1. Whole blot for Western blot images in Figure 6C. Rectangles present areas cropped in Figure 6C.
